# Normal sonographic measurements for kidney dimensions in Saudi adult population: A cross-sectional prospective study

**DOI:** 10.1097/MD.0000000000038607

**Published:** 2024-06-14

**Authors:** Ali S. Alyami, Naif A. Majrashi, Meaad Elbashir, Sarra Ali, Nasser Shubayr, Turkey Refaee, Wael Ageeli, Yahia Madkhali, Ali Abdelrazig, Abdullah A. Althobity, Bandar Alwadani, Qurain Turki AlShammari, Ali M. Hendi

**Affiliations:** a Department of Diagnostic Radiography Technology, Faculty of Nursing and Health Sciences, Jazan University, Jazan, Saudi Arabia; b Department of Radiological Sciences and Medical Imaging, College of Applied Medical Sciences, Majmaah University, Majmaah, Saudi Arabia; c Department of Diagnostic Radiology, College of Applied Medical Sciences, University of Hail, Hail, Saudi Arabia; d Department of Medicine, Jazan University, Jazan, Saudi Arabia.

**Keywords:** height, renal dimensions, sex, ultrasonography

## Abstract

**Background:**

The use of ultrasound-based measurements to determine renal size has proven valuable in the diagnosis of renal function and associated disorders. The dimensions of the abdominal organs are affected by an individual’s body age, height, sex, and weight. The objective of this study was to establish the standard sonographic parameters for renal dimensions and to determine the correlation between body measurements and renal dimensions in a population of healthy adults residing in Jazan City, Saudi Arabia.

**Methods:**

The present study was a prospective study conducted at a single center located in Jazan City from February to August 2022. Ninety-five participants underwent abdominal ultrasonography. The process of measuring renal size through sonography entails the measurement of various dimensions of the kidney, such as renal length, width, and thickness. The demographic information of the participants, including their sex, age, height, and weight, was documented. All analyses were performed using Statistical Package for the Social Sciences v27 software.

**Results:**

The dimensions of the right kidney, specifically the length, width, and thickness, had mean value of 9.79 centimeters (cm), 5.09 cm, and 4.10 cm, respectively. The left kidney had mean dimensions of 10.1 cm, 5.09 cm, and 4.10 cm for length, width, and thickness, respectively. The left kidney was larger than the right kidney. Furthermore, male participants exhibited larger kidney measurements than their female counterparts did. A noteworthy positive correlation was observed between the thickness of the left kidney and sex, whereas no significant correlations were found with age, weight, or height.

**Conclusion:**

The current study revealed that the kidney dimensions were observed to be larger in males as compared to females. The research findings indicate that there is no significant correlation between kidney dimensions and various demographic factors, such as age, height, weight, and sex. In addition, this study provides reference tables for further use.

## 1. Introduction

The kidneys are a vital pair of major excretory organs that play a crucial role in maintaining water balance and electrolytes within the body. In addition, they function as endocrine organs. The measurement of renal size is considered critical because various disorders manifest as kidney reduction or enlargement. While standard anatomy textbooks consider the dimensions, particularly the length and width of an adult kidney, to be 12 centimeters (cm) and 6 cm, respectively, the research literature suggests that these dimensions vary among ethnic groups and are influenced by other factors such as body size, age, sex, and body mass index.^[1–3]^ Medical imaging techniques allow for the examination of internal anatomical structures in individuals and investigation of their movements during regular and abnormal activities. Ultrasonography is a reliable, widely accessible, safe, and affordable modality for imaging kidneys.^[4]^ Furthermore, ultrasonography provides excellent visualization of the anatomical features of the kidneys, including their location and dimensions such as length, width, and thickness. In addition, it enables the detection of focal lesions.^[5]^ This modality is currently used in pediatric and adult populations for the evaluation and follow-up of patients with congenital kidney anomalies, kidney transplants, kidney tumors, chronic kidney disease, renal arterial stenosis, kidney stones, and renal cystic diseases.^[6]^

Renal size can be ascertained by measuring renal length, width, and thickness. Several previous studies have observed a correlation between renal measurements obtained via ultrasound and somatic parameters. Specifically, right and left renal lengths were significantly associated with body mass index (BMI), weight, and body surface area.^[7]^ Renal morphology has been reported to be correlated with body indices, such as gender, age, sex, body weight, height, and BMI.^[1,8,9]^ Therefore, establishing population-specific reference values for the normal kidney is imperative for evaluating any potential alterations within a particular population. However, the literature contains inconsistent reports on the relationship between body indices and renal morphology. For instance, some studies have found negative correlations between age and renal size^[10–12]^ while others have demonstrated no correlations.^[13–15]^

Previous research has been conducted on sonographic measurements of renal size in the Saudi population in different cities.^[16–18]^ However, there is a dearth of studies in Jazan city. Malaria is the most common endemic disease in the world and affects millions of people living in tropical regions of the world making it a serious infectious disease with significant public health implications. Jazan is one of these tropical places, as well as one of the most malaria-prone cities in Saudi Arabia.^[19]^ Malaria can cause disorders in the human body, including in the kidneys. Kidney involvement is common in *P. malariae* and *P. falciparum* infections.

In the current retrospective study, we aimed to establish a reference range for normal renal dimensions in a healthy adult population in Jazan region, and to determine the correlations between body indices and renal sonographic findings.

## 2. Material and methods

This prospective cross-sectional study was conducted at a single center over a period of 7 months. This study aimed to evaluate the dimensions of the kidneys in the Saudi population using ultrasound. The purpose of this study was to examine and establish benchmark values for the ranges of renal dimensions in ae typical young adult Saudi population, and to ascertain their correlation with weight, height, and BMI. The study was conducted in the Diagnostic Radiography Technology Department at Jazan University and was approved by the Standing Committee for Scientific Research at Jazan University (REC-43/02/028). All subjects were informed of the purpose of the study and its potential effects, and all provided written consent.

The study’s inclusion criteria encompassed Saudi participants aged 18 to 45 years who met the following criteria: absence of a personal or family history of renal disease, diabetes mellitus, hypertension, and the absence of any chronic or acute illness. The study’s exclusion criteria were individuals from diverse geographic locations and anomalous ultrasonographic observations that could potentially impact renal size, such as adrenal mass, renal abscess, renal mass, renal stone, renal cyst, renal duplication, hydronephrosis, polycystic kidney disease, acute change (e.g., acute pyelonephritis), diabetic nephropathy, columnar hypertrophy, medullary calcinosis, gouty nephropathy, single kidney, and chronic progressive nephropathy. Furthermore, individuals with a medical history of hemoglobinopathies or anemia, specifically thalassemia and sickle cell anemia, were excluded.

### 2.1. Data collection and ultrasonographic examinations

The baseline data consisted of demographic variables, such as age and sex, as well as physical measurements (weight and height of the participants were measured using the standard anthropometric technique). BMI was calculated using the following formula (BMI = weight (kg)/[height (cm)]^2^). The weight of each participant was recorded in kilograms. The height of the participants was measured in centimeters from the bottom of the feet to the top of the head when standing erect without a hat and shoes.

**Figure 1.**
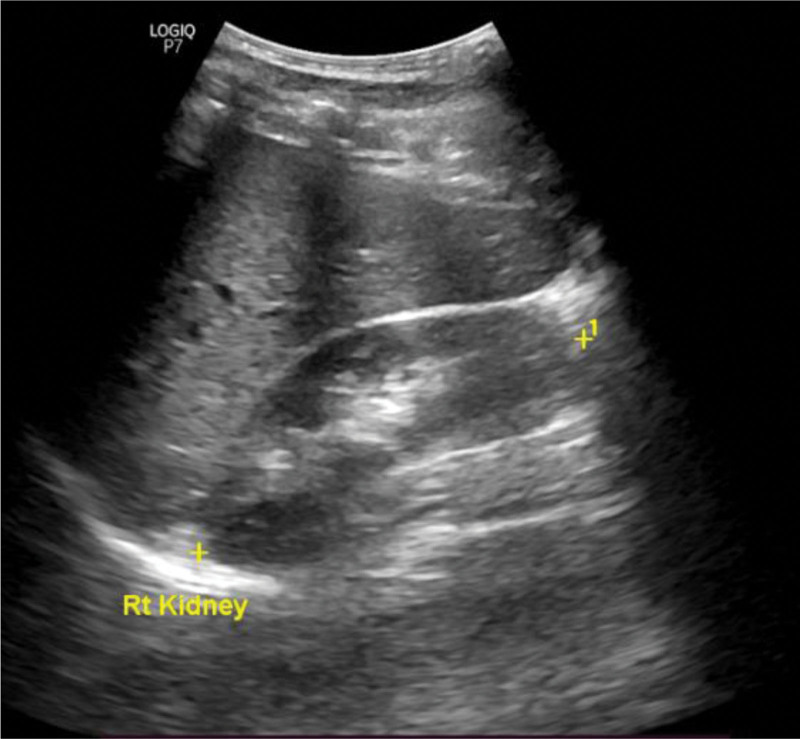
Maximum length of the kidney.

Ultrasound scans were performed using a 3.5 MHz GE ultrasound machine by a well-experienced sonographer with more than 7 years of experience. The participants were scanned in the supine or decubitus position. Kidney length (Fig. 1), width, and thickness were measured in centimeters. The superior and inferior poles were identified and marked on the longitudinal scan of the kidney, and the renal length was taken as the longest distance between the poles using an electronic caliper. In some cases, the patient was requested to raise the ipsilateral arm over the head and take a deep breath to have a good view of both poles. The width and thickness of the kidneys were measured in sections that were perpendicular to the longitudinal axis. The transverse section was located at the level of the vascular hilum of the kidney. The width and thickness were then measured orthogonally in the 2 orientations.

### 2.2. Data analysis

All analyses and data processing were performed using Statistical Package for the Social Sciences software version 27. The main measures of interest, including the kidneys (anteroposterior [AP], length, and width), are summarized in tables of descriptive statistics for all participants, males, and females. The participants were subdivided into age groups based on their age in years; however, because the majority of participants (>80%) were in the age range of 20 to 29 years, no further analyses based on age groups were performed. Because the data were normally distributed (Shapiro–Wilk, *P* > .05, for all variables), we used Pearson correlation to test whether the measures of interest were correlated with demographics such as age, gender, height, weight, and BMI. Additionally, we used a t-test to test whether the measures of interest differed between males and females. Because the rate of organ growth decreases with age (faster growth in very young children), we used analysis of covariance analysis to determine the difference in the organ of interest, adjusting for age and other covariables.

## 3. Results

### 3.1. Demographics

A total of 95 consecutive participants were recruited over 7 months. The study population had a higher proportion of males (57.9%) than comparison to females (42.1%). Their ages ranged from 18 to 45 years old. The age range of 20 to 29 years encompassed a significant proportion of the participants, constituting 80% of the total sample. The mean height, weight, and BMI ranged from 163.8 ± 10.5, 62.6 ± 20.7 kg, and 23.2 ± 6.87 kg/m^2^, respectively. The majority of participants had a normal BMI (mean, 23 ± 6.87). Table 1 summarizes the participants’ demographic characteristics.

**Table 1:** Summary of demographic characteristics.

Demographics	Number	%
Number of participants	95	100
Gender		
Male	55	57.9%
Female	40	41.1%
Age group		
1 (18–19 years)	4	4.2%
2 (20–29 years)	76	80.0%
3 (30–39 years)	11	11.6%
4 (40–45 years)	4	4.2%
**Demographics**	**Mean**	**SD**
Age	23.8	6.36
Height (cm)	163.8	10.5
Weight (kg)	62.6	20.7
BMI (kg/m^2^)	23.2	6.87
Males		
Age	21.6	1.54
Height	171.1	6.60
Weight	68.1	22.3
BMI	23.1	6.87
Females		
Age	26.8	8.83
Height	153.8	5.42
Weight	55.1	15.6
BMI	23.3	6.94

### 3.2. Kidneys dimensions

Table 2 presents an overview of the descriptive statistics for the measurements of the right and left kidneys among all participants, as well as for males and females separately. Table 3 displays a noteworthy correlation only between the left kidney and demographic variables. In this table, length, width, and AP are variables examined by age, height, weight, BMI, and sex. Therefore, it was suggested that the age data be placed in the column, and the length data were put in the raw. We found a statistically significant positive correlation (*P* < .05) between the AP diameter of the left kidney and sex. No statistically significant correlations were observed between the remaining right and left kidney diameters and demographic variables.

**Table 2:** US measurements of the kidney dimensions.

Variables	All participants		Males		Females		*P* value
	Mean	SD	Mean	SD	Mean	SD	*P* for *T*-test
Right kidney (cm)							
Length (cm)	9.79	1.03	9.96	1.21	9.53	.636	**<.001**
Width (cm)	5.09	0.96	5.31	1.08	4.76	.630	**.012**
AP (cm)	4.10	0.80	4.15	.848	4.01	.728	**.013**
Left kidney (cm)							
Length (cm)	10.1	1.02	10.4	.780	9.64	1.14	**<.001**
Width (cm)	5.56	4.48	5.26	.932	5.96	6.84	**.004**
AP (cm)	4.61	0.60	4.80	.570	4.34	.534	**<.001**

Bold values are statistically significant (*P* < 0.05).

**Table 3:** Linear correlations between the dimensions of left and right kidneys and demographic.

Variables	Age		Height		Weight		BMI		Gender	
	r	*P*	r	*P*	r	*P*	r	*P*	r	*P*
Right kidney										
Length	‐.017	.990	.062	.858	.498	.228	‐.532	.511	.178	.256
Width	.070	.646	‐.185	.634	.546	.546	‐.325	.718	.261	.097
AP	.014	.930	.256	.514	.533	.562	‐.369	.685	‐.289	.102
Left kidney										
Length	.227	.126	.057	.872	.473	.583	‐.240	.771	.280	.080
Width	‐.249	.144	.240	.550	‐.582	.558	.648	.495	‐.187	.306
AP	‐.116	.448	.032	.931	‐.196	.826	.446	.559	**.373**	**.023**

Bold values are statistically significant (*P* < 0.05).

### 3.3. Comparison of measures of kidneys dimensions between males and females

Table 2 indicates that there were differences in all metrics of both the left and right kidneys between sexes. The observed differences were statistically significant (*P* < .05) for all metrics. Specifically, females exhibited smaller kidney dimensions than males, with the exception of the width of the left kidney, where females had larger measurements than males. An analysis of covariance was employed to ascertain whether there were noteworthy variations between males and females in these dimensions, after controlling for covariates such as age, height, weight, and BMI.

## 4. Discussion

Normal renal measurements are critical for investigating renal function and disease. It is also significant for the initial diagnosis as well as the subsequent treatment of patients with renal disease to track disease progression.^[20]^ Renal size is subject to variation due to a range of factors such as disease states, anthropometric parameters, and physiological processes. This size offers an approximate indication of the renal function. Chronic renal failure, renal arterial occlusion, and late-stage renal venous thrombosis all cause reductions in size and function.^[21,22]^ The availability of ultrasound imaging across the nation has made it a tool of choice for assessing these metrics. It is still frequently used because it is noninvasive, reproducible, and accessible despite certain limitations such as variances in observers’ skills and interpretation, patient participation, and positioning.^[20]^

Ablett et al^[23]^ evaluated interobserver and intra-observer variability in measures of renal length and discovered that the level of variance was the same regardless of whether the left or right kidney was assessed and whether measurements were taken by one or many sonographers. This indicates that measurements of the normal adult kidney’s bipolar length obtained by sonography are relatively reliable.^[11]^ Most studies have focused on the kidney length. The most often used and practicable measurement in clinical practice is the ultrasonic kidney length (bipolar measurement), which is associated with renal function.^[24]^

The findings of the current study on renal length, width, and thickness in asymptomatic participants were comparable to those reported in previous studies. However, kidney length in the asymptomatic group was much shorter than that in other locations.^[13,25,26]^ It is possible that racial and genetic influences occur among individuals from various nations, depending on body shape, size, and habitus. However, compared to similar groups in different cities in the country, there was no difference in kidney length between our study and the study by Elsayed et al.^[16]^ Elsayed et al found that the mean renal length in the total group was (9.91 cm ± 0.85) on the right side and (10.17 cm ± 0.89) on the left side and with the left side longer.^[16]^

Kidney size was significantly influenced by age. Numerous studies have highlighted the relationship between kidney and demographic size. Emamian et al^[9]^ reported evidence linking kidney dimensions (length, width, depth, and volume) to weight, height, and body surface area, and BMI, while Han and Babcock^[27]^ reported a correlation between kidney length and BMI. According to our study, there was no significant correlation between the kidney size and age. It is possible that the limited number of older participants affected the results of the present study. In addition, demographic characteristics were not correlated with kidney size; however, there was a positive correlation between the left kidney AP diameter and sex. The remaining right and left kidney size and demographic characteristics were not significantly correlated. This agrees with AlShammari et al,^[18]^ who found no remarkable correlation between renal length and sex in young participants in Hail city. However, this contradicts the findings of Saeed et al, who found a constant variation in kidney length between sexes in their children’s participants.^[28]^ One possible explanation for this discrepancy may be the targeted age group.

There were significant differences in all measures of both the left and right kidneys between males and females. This finding is inconsistent with those of previous studies.^[1,13]^ This may be due to the incomparable body weights of the sexes. In addition, the current study found that the mean left renal length, width, and thickness were significantly greater than the right renal dimension. Previous studies have reported similar finding.^[12,25,29]^ This could be because the left renal artery is shorter than the right, causing increased blood flow on the left side, and as a result, the left kidney receives more blood and oxygen than the right kidney. Furthermore, both kidney dimensions were greater in men than in women in our participants’ population, except for the width of the left kidney, which was greater in women than in men. These findings are somewhat consistent with those of previous studies that found men’s kidney diameters to be significantly larger than those of women’s.^[25,30]^ These discrepancies may be due to the use of different radiological methods to estimate kidney dimensions.

The current study had some limitations. First, most of the participants were in the second decade, while fewer participants were from other groups, which may have affected the results. Second, the study was conducted at a single institution, suggesting that multi-center studies may provide some insights owing to the variability of the cases. Third, the ultrasound exams were performed by only one sonographer, although the findings might be equivocal. Therefore, an inter-observer study is necessary. Fourth, our study did not consider variables such as race, culture, ethnicity, or socioeconomic level. Fifth, this study had a small sample size. Further research on a large population and external validation are needed to determine normal values and assess our findings.

## 5. Conclusion

The findings revealed intriguing patterns in renal measurements, emphasizing the significance of considering sex, age, and other demographic factors. Notably, we observed differences between the male and female kidney dimensions, with certain measurements displaying statistically significant variations. The left kidney, in particular, exhibited greater dimensions than the right kidney, contributing to a broader understanding of renal asymmetry.

## Acknowledgments

The authors extend their appreciation to the Deputyship for Research & Innovation, Ministry of Education in Saudi Arabia, for funding this research work through project number ISP23-100.

## Author contributions

**Conceptualization:** Ali S. Alyami, Naif A. Majrashi, Nasser Shubayr, Meaad Elbashir, Wael Ageeli.

**Data curation:** Ali S. Alyami, Naif A. Majrashi.

**Investigation:** Ali S. Alyami, Nasser Shubayr, Meaad Elbashir.

**Methodology:** Ali S. Alyami, Naif Majrashi, Wael Ageeli.

**Resources:** Meaad Elbashir.

**Software:** Turkey Refaee.

**Supervision:** Ali S. Alyami, Naif A. Majrashi.

**Writing – original draft:** Ali S. Alyami.

**Writing – review & editing:** Naif A. Majrashi, Nasser Shubayr, Ali M. Hendi, Ali Abdelrazig, Sarra Ali, Yahia Madkhali, Wael Ageeli, Bandar Alwadani, Abdullah A. Althobity, Qurain Turki AlShammari, Turkey Refaee.
